# *Eimeria stiedae* causes most of the white-spotted liver lesions in wild European rabbits in Cambridgeshire, United Kingdom

**DOI:** 10.1177/10406387211066923

**Published:** 2022-01-24

**Authors:** Diana Bochyńska, Sheelagh Lloyd, Olivier Restif, Katherine Hughes

**Affiliations:** Department of Veterinary Medicine, University of Cambridge, Cambridge, United Kingdom; University of Agricultural Sciences and Veterinary Medicine of Cluj-Napoca, Cluj-Napoca, Romania; Department of Veterinary Medicine, University of Cambridge, Cambridge, United Kingdom; Department of Veterinary Medicine, University of Cambridge, Cambridge, United Kingdom; Department of Veterinary Medicine, University of Cambridge, Cambridge, United Kingdom

**Keywords:** *Calodium hepaticum*, coccidiosis, *Eimeria stiedae*, liver, parasitology, pathology, rabbits

## Abstract

In rabbits, a white-spotted liver can be indicative of one of several disease processes, frequently caused by parasites. To date, the prevalence of white-spotted liver in wild rabbits, *Oryctolagus cuniculus*, in the United Kingdom is undetermined. We evaluated the prevalence and main parasitic etiologies of this entity in a U.K. population of wild rabbits. Wild rabbits (*n* = 87) were shot in Cambridgeshire for population control, and cadavers were donated for research. Postmortem examination was undertaken, including gross and histologic hepatic examination. Macroscopic lesions consistent with white-spotted liver were found in 46 of 87 (53%) rabbits examined; most of these lesions were considered to be mild. For 28 of 46 (59%) rabbits with gross hepatic lesions, an etiologic agent was apparent histologically. *Eimeria stiedae* was detected in 21 of 87 (24%) rabbits, and *Calodium hepaticum* (syn. *Capillaria hepatica*) was detected in 7 of 87 (8%). In the subset of rabbits killed in the summer, there was a significant association between white-spotted liver and juvenile age class. There was also an association between white-spotted liver caused by *E. stiedae* and juvenile age class. When restricting analysis to rabbits with white-spotted liver caused by *E. stiedae* and submitted in the summer, both juvenile age class and female had significant effects. *E. stiedae* and *C. hepaticum* can be transmitted to pet lagomorphs via contaminated vegetation, and to humans in the case of the latter, which demonstrates the importance of monitoring the prevalence of these parasitic diseases in wild rabbits.

European rabbits (*Oryctolagus cuniculus*) are common pet animals worldwide and are increasing in popularity in many countries.^8^ The population of pet rabbits in the United Kingdom is estimated at ~900,000 animals, with ~2% of households owning such pets.^24^ The population of wild rabbits in the United Kingdom is estimated at ~36,000,000 animals.^16^ With the high numbers of both wild and pet populations, there is a risk of wild rabbits being a reservoir of infectious agents for pet rabbits. For example, when considering leporine hepatic diseases, contamination of grass and other plants with *Eimeria stiedae* oocysts may lead to transfer of the agent from wild to pet rabbits.^11^ There is also a potential risk of zoonotic spread of organisms such as *Calodium hepaticum* (syn. *Capillaria hepatica*).^21^

In rabbits, a macroscopically white-spotted liver can be indicative of one of several disease processes that cause similar gross hepatic changes, consisting of small 1–2-mm, white-or-cream focal lesions scattered throughout the hepatic parenchyma. There are numerous parasitic causes of white-spotted livers in lagomorphs, including *E. stiedae*,^17^ *Cysticercus pisiformis*,^1^ and *C. hepaticum*^2^; bacterial agents that can cause hepatic abscesses include *Yersinia pseudotuberculosis*^25^ and *Francisella tularensis*.^13^

The organism that is perhaps most typically associated with white-spotted livers in rabbits is *E. stiedae*.^25^ *E. stiedae* are coccidian parasites found in both wild and pet rabbits.^17^ Hepatic coccidiosis is potentially fatal, and occurs mostly in young, weanling rabbits, resulting in poor weight gain.^25^ The disease is spread by the fecal-oral route. Ingested sporocysts develop into sporozoites, which penetrate the duodenal mucosa to migrate to the mesenteric lymph nodes, where they can be found within 12 h after ingestion of the sporocysts. The parasites undergo hepatic migration via mononuclear cells within lymphatics. In the liver, the parasite colonizes the biliary epithelium in which schizogony, gametogony, and oocyst formation occur. Oocysts are released to the bile ducts, are transported to the intestines, and are passed subsequently to the environment via the feces.^3^ There are few studies examining the prevalence of hepatic coccidiosis worldwide: the prevalence was reported as 31% among farmed rabbits in Indonesia (*n* = 750)^10^ and 11.5% in farmed rabbits in Kenya (*n* = 302).^19^

*C. hepaticum* is a nematode parasite that colonizes the liver. *C. hepaticum* can cause an important and potentially fatal zoonosis.^21^ The female nematodes release ova into the hepatic parenchyma, and these eggs contaminate the environment if the host dies or is killed and is subsequently eaten. Humans, rodents,^20^ or other lagomorphs can be infected orally by ingesting food contaminated with eggs, or by direct ingestion of contaminated soil.

Another cause of white-spotted liver in rabbits is *C. pisiformis*, which is the larval stage of *Taenia pisiformis*. The definitive hosts for this cestode in the United Kingdom are dogs and foxes, with rabbits an intermediate host. Tapeworm segments packed with eggs are shed in the feces of dogs and foxes, and the eggs are ingested from the pasture by rabbits. The larval stage migrates from the small intestine, and for a time in the liver, before encysting in the peritoneal cavity and sometimes in the liver.^26^ The life cycle is completed when an infected rabbit is eaten by the definitive host. The prevalence of *C. pisiformis* in a slaughter farmed rabbit population in Poland was estimated to be 4.47% (*n* = 274).^23^

Thus, although several potential etiologies for the leporine white-spotted liver have been documented, for the population of wild rabbits in the United Kingdom, the relative contribution of each parasitic etiologic agent to the gross presentation of a white-spotted liver is not clear. Our goal was therefore to catalog the occurrence of white-spotted livers in a population of wild rabbits shot for population control, to present a system for classification of the severity of the lesions, and to examine the underlying causes of the disease process in these animals.

## Materials and methods

### Study population

Wild European rabbits (*n* = 87) were examined. All rabbits were shot as part of a population control program between July 2016 to February 2017 in Cambridgeshire, United Kingdom and donated for research and teaching purposes to the veterinary anatomic pathology service of the Department of Veterinary Medicine, University of Cambridge. The study was approved by the Ethics & Welfare Committee of the Department of Veterinary Medicine, University of Cambridge (reference CR240).

### Gross and histologic examination

**Figures 1–5.**
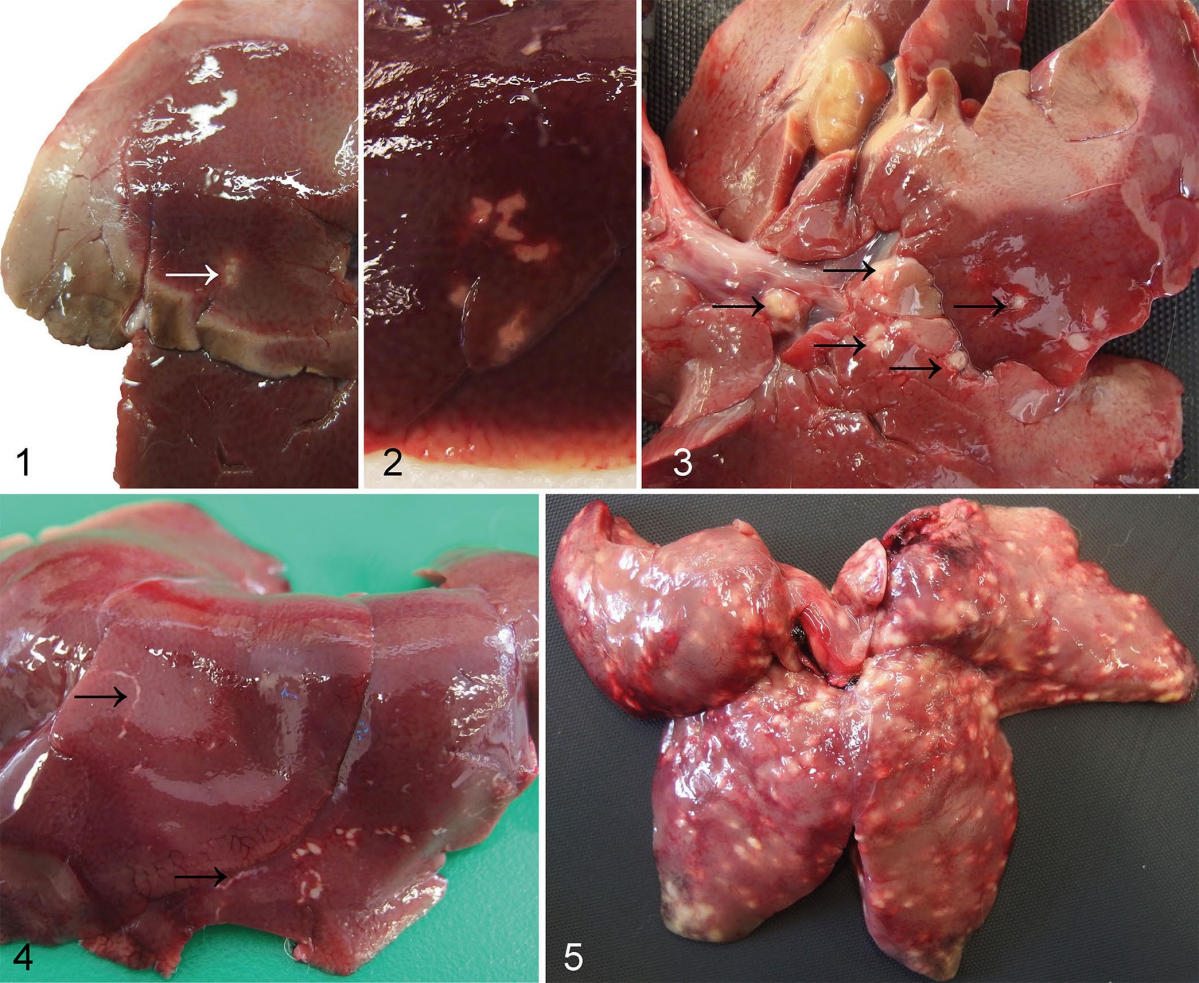
Grading system for macroscopic lesions of white-spotted liver in the rabbit. **Figure 1**. Minimal lesion: the hepatic parenchyma is expanded by a single cream focus ≤3-mm diameter (arrow). **Figure 2**. Mild lesion: >3 but <10 cream-to-white foci ≤3-mm diameter. **Figure 3**. Moderate lesion: <10 cream-to-white foci, >3-mm diameter (arrows). **Figure 4**. Moderate linear or serpiginous lesion: >10 but <30 cream-to-white, predominantly serpiginous foci, ≥5 mm in length (arrows). **Figure 5**. Marked lesion: the hepatic parenchyma is expanded by numerous cream-to-white bosselated foci.

Rabbit cadavers were stored at 4°C, and gross postmortem examination was carried out usually within 48 h and always within 120 h. Rabbit cadavers were weighed prior to commencement of the postmortem examination. Livers were dissected and weighed, except in cases of agonal rupture. All postmortem examinations were carried out by a single ACVP board-certified pathologist (K. Hughes) following standard lagomorph postmortem examination procedures. Standardized criteria were used for gross classification of white-spotted hepatic lesions (Table 1; Figs. 1–5).

**Table 1.** Descriptive criteria for gross changes associated with white-spotted liver in the rabbit and number of cases with an established definitive etiology for lesions of each criterion.

Classification	Criteria: no. of cream-to-white foci	Overall prevalence of lesion	Lesions of this macroscopic severity caused by *E. stiedae*	Lesions of this macroscopic severity caused by *C. hepaticum*
Minimal (Fig. 1)	≤3, ≤3-mm diameter	14 of 46 (30%)	5 of 14 (36%)	0 of 14 (0%)
Mild (Fig. 2)	3 to ≤10, ≤3-mm diameter	18 of 46 (39%)	10 of 18 (56%)	2 of 18 (11%)
Moderate (Fig. 3)	>10 and <30, <3-mm diameter; or ≤10 foci >3-mm diameter	9 of 46 (20%)	4 of 9 (44%)	4 of 9 (44%)
Marked (Fig. 5)	>30 foci, any size	2 of 46 (4%)	2 of 2 (100%)	0 of 2 (0%)
	Criteria: no. of linear or serpiginous lesions, ≥5 mm long	Overall prevalence of lesion	Lesions of this macroscopic severity caused by *E. stiedae*	Lesions of this macroscopic severity caused by *C. hepaticum*
Minimal, mild, marked	≤3; >3 but <10; >30, respectively	0 of 46 (0%)	NA	NA
Moderate (Fig. 4)	>10 but <30	3 of 46 (7%)	0 of 3 (0%)	1 of 3 (33%)

NA = not applicable.

Grossly evident hepatic lesions were photographed and sampled, and if no lesions were detected macroscopically, representative hepatic parenchyma was collected during the postmortem examination. Liver tissue and samples from any other lesions in other organ systems were fixed for 24 h in 10% neutral-buffered formalin, processed routinely, sectioned at 5 µm, and stained with H&E.

Hepatic sections were assessed subjectively, and histologic lesions classified by one investigator (D. Bochyńska) with guidance and oversight of a second investigator (K. Hughes).

### Data handling and statistical analyses

Juvenile rabbits were defined as <1,249 g, and these animals were considered to be younger than ~130 d.^14^ Results were recorded in Excel and then transferred to Prism v.9.1.0 (GraphPad) for statistical analyses. We carried out a binomial logistic regression analysis with a gross diagnosis of white-spotted liver (of any degree of severity) as the binary response variable, and sex (male/female), season (summer/autumn), and age group (adult/juvenile) as explanatory variables. We then applied backward stepwise elimination (using the step function in R, which compares models based on the Akaike Information Criterion) to identify explanatory variables, with significant effects at the 0.05 level. Because of the substantial differences in numbers and age distributions of rabbits between seasons, we then repeated the analysis just for the animals killed in the summer, which formed the largest and most homogeneous subset. We then carried out the same analysis of potential risk factors for the presence of *E. stiedae*, which was the most common etiology for white-spotted liver in our data set. We report the significant variables present in the final models and their odds ratios with 95% CIs for prevalence estimates.

## Results

### Study population

Thirty-five of 87 (40%) rabbits were females; 52 of 87 (60%) were males. Most of the rabbits (63 of 87; 72%) were adults. The mean and median body masses were 1.63 kg and 1.64 kg, respectively (range: 1.28–2.18 kg) for adult rabbits, and 1.04 kg and 1.05 kg, respectively (range: 0.64–1.24 kg) for juveniles. The weight of one animal was not recorded. Of the adult population sampled, 25 of 63 (40%) were females and 38 of 63 (60%) were males. Four females were lactating; none were pregnant. Fifty-nine of 87 (68%) animals were submitted in U.K. meteorologic summer (June–August), 22 of 87 (25%) in the autumn (September–November), and the remaining 6 of 87 (7%) in the winter (December–February). Of the 23 juveniles, 21 were submitted in the summer. No animals were shot in the spring.

### Gross lesions of white-spotted liver

**Figure 6.**
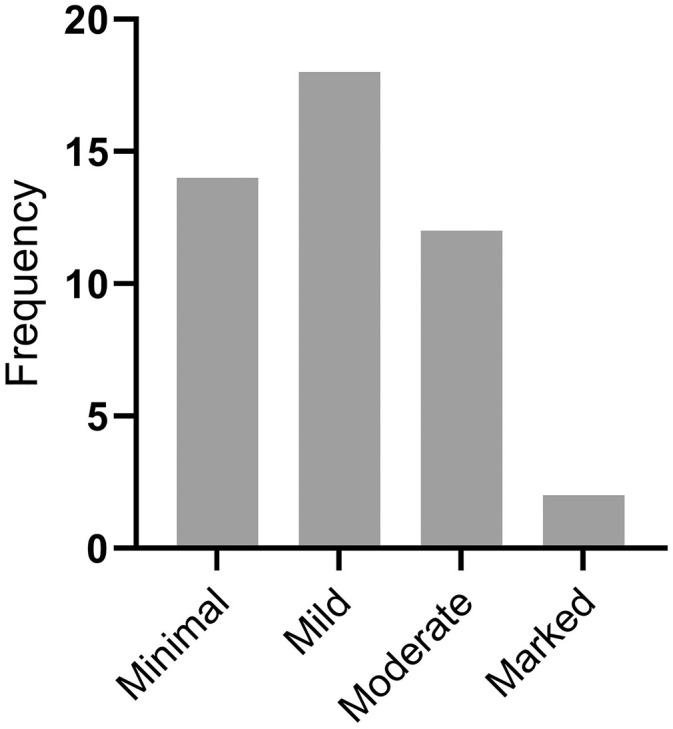
Frequency histogram of the distribution of severity of lesions among 46 rabbits with detected white-spotted liver lesions.

Of the rabbits examined, 46 of 87 (53%) had gross lesions consistent with white-spotted liver. These hepatic lesions were classified grossly as minimal in 14 rabbits, mild in 18 rabbits, moderate in 12 rabbits, and marked in 2 rabbits (Fig. 6). Of the rabbits exhibiting moderate white-spotted gross lesions, 3 had distinctive linear or serpiginous lesions (Fig. 4). Of the 3 rabbits with distinctly linear or serpiginous gross foci, 1 rabbit had a diagnosis of *C. hepaticum* infection; an etiologic agent was not identified in the other 2 rabbits.

### Histologic lesions associated with white-spotted liver

**Figures 7, 8.**
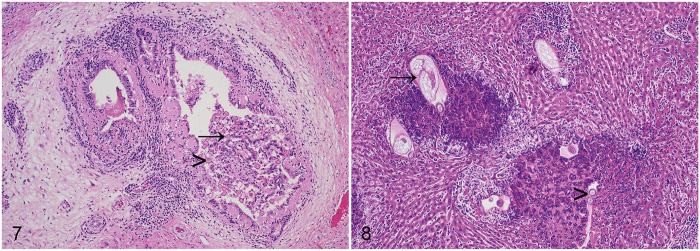
Parasitic etiologies of white-spotted liver in the rabbit. **Figure 7**. The hyperplastic epithelium of an ectatic bile duct is expanded by macrogamonts (arrow) and oocysts (arrowhead) consistent with *Eimeria stiedae*. The bile duct is surrounded by fibrous tissue and a predominantly mononuclear inflammatory infiltrate. H&E. **Figure 8**. Numerous viable and degenerate heterophils and fewer lymphocytes and plasma cells surround nematode profiles consistent with *Calodium hepaticum* (arrow). Eggs are also present (arrowhead). H&E.

For 28 of 46 (59%) of the rabbits with gross hepatic lesions, an etiologic agent was apparent histologically. Overall, *E. stiedae* was detected in 21 of 87 (24%) rabbits. Infection with *E. stiedae* was diagnosed on the basis of ectasia of the bile ducts resulting from epithelial hyperplasia, characteristic papillary luminal projections, large numbers of intraepithelial gametocytes and oocysts, as well as portal fibrosis, as reported previously (Fig. 7).^4,6^ Macrogametes were spherical, 20–50-µm diameter, with peripherally located intracytoplasmic eosinophilic granules, a centrally located nucleus, and a prominent nucleolus. Microgametes were also spherical, 15–25-µm diameter, with peripheral intracytoplasmic basophilic granules. The nonsporulated oocysts were oval, 20–40-µm diameter, with thick walls, granular cytoplasm, and eosinophilic nuclei. Most cases of hepatic coccidiosis were classed grossly as mild infections (11 of 21 of the rabbits with hepatic eimeriosis; 52%). Notably, however, 2 of 21 (10%) of the animals with hepatic coccidiosis had marked lesions grossly (Fig. 5).

*C. hepaticum* was detected in 7 of 87 (8%) rabbits. Infection with *C. hepaticum* was diagnosed on the basis of characteristic histologic lesions comprising parenchymal granulomas with centrally located 40 × 60-µm eggs with a thick shell and prominent bipolar opercula (Fig. 8).^15^ Although some granulomatous lesions affected biliary foci, overall distribution was random, and not focused particularly on the biliary epithelium, consistent with the parasitic life cycle. Of the 7 histologically confirmed cases of *C. hepaticum*, 4 of the 7 (57%) cases had moderate histologic changes, and the remaining 3 of 7 (43%) had marked histologic changes. Only one rabbit infected with *C. hepaticum* was a juvenile.

For 18 of the 46 (41%) rabbits exhibiting gross hepatic lesions, no etiologic agent was detected in the tissue sections examined histologically. In these animals, the lesions were generally composed of predominantly minimal-to-mild, but in some cases moderate, portal lymphoplasmacytic hepatitis, with a few small midzonal granulomas. The midzonal granulomas were irregular and consisted of necrotic centers that were surrounded by a moderately thick layer of epithelioid macrophages and fibroblasts and by a variably thick layer of lymphocytes and plasma cells. No parasites were identified histologically in animals that lacked gross lesions.

### Risk factors for the occurrence of white-spotted liver

The occurrence of white-spotted liver appeared to be lower in adults (30 of 63; 48%) than in juveniles (16 of 23; 70%) and higher in the summer than in the autumn and winter (34 of 59 [58%] vs. 12 of 28 [43%]), although neither factor reached statistical significance in our logistic regression model. Because all but 2 juveniles were submitted in the summer, we repeated the analysis to test for potential effects of age and sex on white-spotted liver prevalence among the subset of rabbits killed in the summer (*n* = 59); there was a significant association with age (logistic regression *p* = 0.037, odds ratio of 0.28 for adults; 95% CI: 0.079, 0.88) and no significant association with sex (*p* = 0.7).

### Risk factors for the presence of *E. stiedae*

Among the rabbits examined, hepatic eimeriosis was more prevalent in juveniles (10 of 23; 43%) than adults (11 of 63; 17%), and more prevalent in females (12 of 35; 34%) than in males (9 of 52; 17%). In our logistic regression model, only age had a statistically significant effect (*p* = 0.017, odds ratio 0.21 for adults; 95% CI: 0.06, 0.68). For rabbits submitted in the summer, both age (*p* = 0.013) and sex (*p* = 0.031) had significant effects; the odds ratios were 0.19 (95% CI: 0.05, 0.67) for adults and 0.24 (95% CI: 0.06, 0.85) for males.

### Liver weights of rabbits with no evidence of hepatic disease

No gross hepatic lesions were observed in 41 of 87 (47%) rabbits. Excluding the rabbits that had agonal hepatic rupture, liver mass data were available for 29 adult rabbits with no gross hepatic lesions. Both the median and mean liver masses as a percentage of body mass for this group were 2.97% (range: 2.19–4.16%).

## Discussion

We found that gross lesions consistent with white-spotted liver are common in wild rabbits in Cambridgeshire; 46 of 87 (53%) rabbits were affected. We identified 2 parasitic etiologies for the gross lesions of white-spotted liver, namely *E. stiedae* in 21 of 87 (24%) rabbits examined, and *C. hepaticum* in 7 of 87 (8%) rabbits. We found no cases of *C. pisiformis* despite careful macroscopic inspection of the liver and mesenteries for cysts, and histologic evaluation of hepatic tissue sections. No other species of parasite was detected in the liver. Two animals did have *Coenurus serialis* (larval stage of *Taenia serialis*) cysts in the subcutis or musculature, but it is not anticipated that *C. serialis* would cause hepatic lesions during its migration.

The finding that 21 of 87 (24%) rabbits in our study were affected by hepatic *E. stiedae* is in line with data collated from wild *O. cuniculus* sampled in other countries. A study examining fecal samples from rabbits trapped in Western Australia revealed a prevalence of 25.8%,^12^ whereas in France the prevalence was 4–21%, depending on the region.^9^ Given that the prevalence of *E. stiedae* in France varies by region, further studies may be indicated to generate large-scale prevalence data covering more geographic regions of the United Kingdom.

In a number of cases of white-spotted liver, no etiologic agent was identified, and these cases tended to have nonspecific chronic mononuclear-to-granulomatous lesions histologically. Microbiologic examination of livers not apparently affected by parasites was not performed, and the cause of these lesions remains unknown. It is likely that some of these cases were the result of chronic resolving hepatitis caused by a previous parasitic infection, or that parasites were present but not detected in the histologic sections examined. Although microbiologic examination of the livers was not undertaken, infection with bacterial pathogens was considered unlikely because the histologic lesions were not consistent with a bacterial etiology, and the animals were not found dead or moribund.

The sampling method adopted in our study, using animals shot for population control, means that animals that were clinically moribund, either because of hepatic eimeriosis or other causes, were considerably less likely to have been sampled. Nonetheless, nonrandom, active sampling is a recognized technique in the investigation of wildlife diseases and provides useful information, particularly given the difficulties associated with identification and recovery of diseased carcasses.^18^

An additional limitation of our study is that no animals were sampled during the meteorologic spring because of the schedule of the pest controller. The majority of the animals were sampled during the meteorologic summer, which likely influenced the results. An historic paper reported that prevalence of hepatic coccidiosis increases in the spring and summer in wild rabbits as a result of a higher proportion of juvenile susceptible individuals.^22^ Consistently, we observed a significant association between age and the occurrence of white-spotted liver, with a lower prevalence among adults than juveniles in the subset of rabbits killed in the summer. This association was also true when considering only cases of hepatic eimeriosis irrespective of season. When restricting our analysis to the rabbits submitted in the summer, both age and sex had significant effects on odds ratios for hepatic eimeriosis.

The sampling methodology necessitated that our study was designed as an exploratory analysis based on a convenience sampling technique. Therefore, it is possible that our study is under-powered for detection of association between gross manifestations of white-spotted liver and other parameters.

The hepatic lesions of white-spotted liver were variable in both severity and gross presentation, although most were small round foci or bosselated lesions. In addition, a small number of livers with linear or serpiginous lesions were recognized. Linear lesions of white-spotted liver have been described, particularly in relation to hepatic eimeriosis.^3^ However, we detected linear or serpiginous lesions in association with *C. hepaticum* infection, suggesting that there is not a definitive association between linear or serpiginous lesions and a particular type of hepatic parasite. Therefore, it appears that sub-categorization of the lesions of white-spotted liver according to whether linear or serpiginous foci are present is not necessarily informative in the determination of underlying etiology. It should be noted, however, that only 3 livers with linear or serpiginous white foci were present within our study cohort.

*E. stiedae* is an important, commonly seen, protozoal organism that was definitively detected in 24% of the rabbits in our study. This finding adds weight to the assertion that wild rabbits may be a source of infection for pet rabbits kept outside or provided with cut grass and vegetation that may have been accessed by wild rabbits.^11^ *E. stiedae* has been documented to cause clinical disease in pet rabbits, that in severe cases may develop clinical signs, such as diarrhea, anorexia, and lethargy.^17^ Although most rabbits in our study had lesions of lesser magnitude and may have been infected subclinically, at least one animal had severe lesions similar to those recently reported in pet rabbits.^17^ Interestingly, other investigators have demonstrated reduced body mass in wild rabbits infected with *E. stiedae*,^14^ and it is possible that the infections noted in our study may have impacted body mass even if most of the rabbits were otherwise infected subclinically. Although it is suggested classically that wild rabbits may be a source of infection for domestic lagomorphs in suburban environments, the converse may also be true in the case of pet rabbits kept outside.

Our data suggest that, in the geographic region sampled, infection with *C. hepaticum* is a less common cause of white-spotted liver in rabbits than *E. stiedae. C. hepaticum* has been reported in a European brown hare (*Lepus europaeus*) in the United Kingdom,^2^ and is likely a notable cause of white-spotted livers in rabbits. *C. hepaticum* has also been described in numerous mammalian and avian species.^7,15^ Indeed, the gross and microscopic lesions of *C. hepaticum* infection in the rabbits in our study are strikingly similar to those described in wild urban Norway rats (*Rattus norvegicus*).^20^ Although it is possible that rats may have been a source of infection for the rabbits in our study, no information is available regarding the habitat in which the rabbits were shot, and the potential proximity to rats. Environmental contamination with parasites from wildlife species surviving in urban environments has been demonstrated to impact other potential wildlife host species and human exposure to zoonotic diseases.^5^ Infection of wild rabbits in Cambridgeshire with *E. stiedae* and *C. hepaticum* has the potential for transmission to pet lagomorphs, and to humans in the case of the latter nematode.

Although examination of gastrointestinal or fecal samples is frequently undertaken to detect *E. stiedae* in rabbits,^9,12^ we adopted a different strategy utilizing gross and microscopic investigation of white-spotted liver lesions. We have thus provided a novel framework of descriptive criteria for the gross changes associated with white-spotted liver in the rabbit. Our data provide evidence that both *E. stiedae* and *C. hepaticum* are parasitic etiologies of white-spotted liver in wild *O. cuniculus* in the United Kingdom. We found that distinctive linear or serpiginous lesions are not definitively associated with one particular parasite, underlining the requirement for histology in addition to macroscopic assessment.
